# Acalculous cholecystitis associated with levodopa-carbidopa intestinal infusion therapy: A case report

**DOI:** 10.1016/j.prdoa.2022.100150

**Published:** 2022-07-01

**Authors:** Viviana Torres, Guillermo González-Ortega, Antoni Suárez, Alicia Garrido, Ana Cámara, Yaroslau Compta, Francesc Valldeoriola

**Affiliations:** a Parkinson’s Disease and Movement Disorders Unit, Neurology Service, Institut de Neurociencies, Hospital Clínic of Barcelona, Catalonia, Spain; b Parkinson's Disease & Movement Disorders Unit, Neurology Service, Hospital Clínic I Universitari de Barcelona; IDIBAPS, CIBERNED (CB06/05/0018-ISCIII), ERN-RND, Institut Clínic de Neurociències (Maria de Maeztu Excellence Centre), Universitat de Barcelona. Barcelona, Catalonia, Spain; Lab of Parkinson Disease and Other Neurodegenerative Movement Disorders: Clinical and Experimental Research; Department of Neurology, Hospital Clínic de Barcelona, Institut D'Investigacions Biomèdiques August Pi I Sunyer (IDIBAPS), Institut de Neurociències, Universitat de Barcelona, Barcelona, Catalonia, Spain

## Abstract

Continuous intra jejunal infusion of levodopa-carbidopa intestinal gel (LCIG) is one of the primary therapies for improving advanced Parkinson's disease symptoms. Placement of the jejunal catheter through the abdominal wall for drug administration requires a percutaneous interventional procedure called percutaneous endoscopic gastrostomy (PEG). PEG is considered a safe and straightforward procedure, and it is performed very commonly in clinical practice. In the context of LCIG treatment, severe adverse events have been identified, such as intestinal bleeding and acute abdomen [1], but acute acalculous cholecystitis (AAC) has never been reported.

## 1 Case report

We present a 66-year-old patient with a medical past history relevant for hypertension, type 2 diabetes mellitus, dyslipidemia, sleep apnea, and obesity. He was diagnosed with Parkinson's disease at age 51, being the initial symptom tremor involving his left upper extremity. Initially, he was treated with levodopa and dopaminergic agonists (pramipexole) with a good clinical response. After five years, he developed motor fluctuations, and generalized dyskinesias. Other complications of therapy included binge eating and hallucinations related to the use of dopamine agonists. Mild cognitive impairment was also observed. At this moment, he received adaily equivalent levodopa dose of 1600 mg. Besides, he also suffered mental depression and anxiety requiring specific treatment with duloxetine and clonazepam.

The progressive clinical worsening due to motor complications and side effects of medications, led us to consider the use of intestinal levodopa intraduodenal infusion.

The patient scored III at the Hoen&Yahr scale in the “on” period and IV in the “off” period. He had mild cognitive impairment scoring on Montreal Cognitive Assessment (MoCA) 23/30 at hospital admission. Initial UPDRS III scored 55. Blood tests before the procedure did not disclose any significant abnormalities.

The nasojejunal test improved postural stability, motor fluctuations, and gait. PEG placement was performed two days after. It took one attempt without success to perform the procedure because of great solid content in the stomach, requiring fasting for more than 24 h, and the use of laxatives. The next day, the operation for PEG catheter placement took place without complications; he received antibiotic prophylaxis with meropenem and linezolid, an anesthetic plan with desflurane, fentanyl, and rocuronium, and postoperative pain management with methadone.

On the first postoperative day, the patient developed mild abdominal pain surrounding the PEG incision area. Initially, he presented diminished hydro-air noises with no inflammatory or peritoneal irritation signs. An abdominal X-ray showed a fecal mass; he received intestinal enemas with an improvement in symptoms. Three days later, the patient developed an acute right-sided abdominal pain that required analgesia with methadone and tramadol. Blood examinations showed severe inflammatory response and elevation of liver enzymes. An abdominal CT was indicated. It showed acute cholecystitis signs with inflammatory changes in adjacent fat and the transverse colon. Thus, an urgent cholecystectomy was performed; areas of gallbladder necrosis were observed in the absence of gallstones.

The patient restarted levodopa infusion the next morning. Ten days after admission, cholecystitis symptoms resolved, motor fluctuations improved; dyskinesias and psychiatric symptoms disappeared; l gait and stability improved leading to a partially recovered independence for daily living activities.

## 2 Discussion

Acute acalculous cholecystitis (AAC) is defined as an inflammation of the gallbladder in the absence of associated gallstones. The pathogenesis of the disease is not entirely clarified, although biliary stasis and ischemia are postulated as the main conditions producing gallbladder inflammation [2].

AAC occurs mainly in systemic processes, severe trauma, or after major (cardiac, aortic) or gastrointestinal surgeries, but is also associated with other conditions that can induce a failure in the gallbladder microcirculation (such as diabetes mellitus, congestive heart failure, etc.). Furthermore, the postoperative period after surgeries may aggravate bile stasis by prolonged fasting, hypovolemia, mechanical ventilation, total parenteral nutrition, ileus, and the use of opioids increased intraluminal bile duct pressure [2].

Complications of the bile duct after LCIG have been previously described in a patient with Parkinson's [5].

Multiple gastrointestinal problems secondary to parasympathetic denervation of the digestive tract are common in patients with Parkinson's disease. Recently, some studies using cholescintigraphy techniques have described biliary emptying dysfunction [3], the increase in bile acids at the intestinal level compared to the healthy population, or the greater volume of the fasting gallbladder [4]. The slowing of bile emptying with other procedural related factors such as prolonged fasting, diabetes mellitus, associated vascular and dysautonomic disturbances and the mechanical injury during catheter placement could predispose to the development of AAC in our patient.

In summary, this case illustrates the need for imaging evaluation to rule out gallstones in PD patients previous to the implementation of this therapy; furthermore, given that biliary emptying disturbances are a risk factor for ACC, careful evaluation of any possible precipitating factors has also to be considered before LCGI.

## Declaration of Competing Interest

The authors declare that they have no known competing financial interests or personal relationships that could have appeared to influence the work reported in this paper.
